# Comparison of Post-Operative Outcomes of Right Colectomy between Crohn’s Disease and Adenocarcinoma of the Right Colon: A Retrospective Cohort Study

**DOI:** 10.3390/jcm13102809

**Published:** 2024-05-10

**Authors:** Naama Bursztyn, Edden Slomowitz, Dan Assaf, Ehab Haj Yahia, Ilan Kent, Nir Wasserberg, Shmuel Avital, Ian White

**Affiliations:** 1 School of Medicine, Tel Aviv University, Tel-Aviv 69978, Israel; 2 Internal Medicine A, Shamir Medical Center, Be’er Ya’akov 70300, Israel; 3 Colorectal Unit, Department of Surgery, Beilinson Hospital, Rabin Medical Center, Petach-Tikva 49100, Israel; 4 Department of Surgery, Sheba Medical Center, Tel-Aviv 39040, Israel; 5 Department Surgery B, Meir Medical Center, Kfar Saba 44281, Israel

**Keywords:** Crohn’s disease, colorectal cancer, colectomy, post-operative complications, bowel/intestinal/gastrointestinal surgery

## Abstract

(1) **Background:** Crohn’s disease (CD) and right-sided colorectal-carcinoma (CRC) are two common indications for right colectomies. Many studies have tried to identify risk factors associated with post-operative complications for both CD and CRC. However, data directly comparing the outcomes of the two are sparse. This study aims to compare the 30-day post-operative outcome after a right colectomy for CD versus CRC. Its secondary aim is to identify the factors associated with these outcomes for each group. (2) **Methods:** A retrospective cohort study of 123 patients who underwent a right colectomy for CD or CRC in a single institution between March 2011 and March 2016. (3) **Results:** There were no significant differences between the groups when comparing the overall complication rate, the median Clavien–Dindo score, reoperation rates and the length of hospitalization. The leak rate was higher in the Crohn’s group (13.95% (6/43) vs. 3.75% (3/80)), *p* = 0.049), although the stoma rate was the same (4/43 9.5%; 7/80 9.9%). (4) **Conclusions:** This study has shown that post-operative complication rate is similar for right colectomy in CD and CRC. However, Crohn’s disease patients did have a higher leak rate.

## 1. Introduction

Morbidity following surgery due to Crohn’s disease may be worse than similar surgeries performed due to right-sided colon carcinoma [1,2,3,4,5,6]. However, the literature directly comparing the two groups for their post-operative outcomes is sparse [5]. Colon carcinoma is the third most common cancer, and approximately one-third of all colon carcinomas are located in the right colon [7]. Right colectomy is the standard treatment for early-stage carcinoma of the right colon. Crohn’s disease is a chronic inflammatory bowel disease with the potential to involve any part of the gastro-intestinal tract, including peri-anal disease. The most commonly involved segments in Crohn’s disease are the distal ileum, ileo-cecal valve and cecum [5,8]. Historically, the mainstay of therapy for Crohn’s disease has been medical management, including corticosteroids and a wide compliment of drugs modulating the immune system, such as tumor necrosis factor inhibitors, while surgery is reserved for cases where there are complications or failure of medical treatment [8,9,10].

However, there have been some recent studies [11] which have challenged this paradigm and advocated for upfront surgery at the time of diagnosis. Additional studies are needed to fully elucidate the efficacy of this approach.

A review of the literature shows that the overall complication rate after colonic resection for right-sided colon carcinoma ranges between 22% and 29% [7,12,13]. Meanwhile, the reported complication rate after colonic resection for Crohn’s disease is around 30% [3]. The reported incidence of anastomotic leak (AL) after colorectal procedures varies, with some studies reporting an anastomotic leak rate of 3–6.4% [7,12], while others report a leak rate as high as 8.5% [7,14].

Previous studies have found the following factors to be associated with worse post-operative outcomes for both Crohn’s disease and right-sided colon carcinoma: advanced age [7,12,14,15], male gender [7,12,14,15,16,17], American Society of Anesthesiologists (ASA) score ≥3 [14,16,18], smoking [10,16,19], alcohol use (≥35 drinks per week) [16,19], anemia [9,10], malnutrition (presented as hypoalbuminemia or low total protein concentration) [4,7,9,10,15,16,17,20], leukocytosis [4,10,16], steroid use [4,10,16,20], complicated disease (bowel perforation or obstruction) [4,14], emergency surgery [9,14,16,20], open surgery [7,21] and perioperative blood transfusions [16,19,20].

Disease-specific risk factors for right-sided colon carcinoma include stage II cancer [15], TNM advanced T stage [15] and extensive resection [14]. For Crohn’s disease, penetrating disease (i.e., presence of intra-abdominal abscess or fistula at the time of surgery) [9,10] is a risk factor. Whether immunomodulators or biological treatments (anti-TNFα therapy) increase the risk of post-operative complications in Crohn’s disease patients is still a matter of debate [4,5,9,10,22,23,24,25,26,27,28].

This study aims to directly compare the 30-day post-operative outcomes after a right colectomy for Crohn’s disease versus adenocarcinoma of the right colon. Its secondary aim is to identify the factors associated with these outcomes for each individual group.

## 2. Materials and Methods

### 2.1. Study Design

We conducted a retrospective cohort study on patients who underwent a right colectomy for Crohn’s disease or adenocarcinoma of the right colon (stages 1–3) in the Department of Surgery B in Meir Medical Center, a large-volume academic colorectal referral center in Israel, between March 2011 and March 2016. Data were taken from a prospectively collected database that consists of all patients aged sixteen to eighty years old, who underwent any type of colonic surgery in the Department of Surgery B in Meir Medical Center.

Thirty-day post-operative complications were classified using the Clavien–Dindo classification [29] and compared using diagnosis (Crohn’s disease vs. right-sided colon carcinoma). The data collection as well as assessment of the severity of complications and their classification by the Clavien–Dindo score were performed by an impartial medical student who underwent specific training before the commencement of data acquisition. The student was not directly affiliated with the Department of Surgery B in Meir Medical Center.

This study met the guidelines outlined in the Declaration of Helsinki. This study was approved by the Institutional Review Board (IRB) of Meir Medical Center (IRB #0119-17-MMC). Due to the minimal-risk nature of this study, the need for informed consent was waived by the IRB. Patients’ confidentiality was kept through data collection and analysis by replacing protected personally identifiable information with research identification codes (ID codes).

Collected data included demographic and anthropomorphic details, medical and surgical history, indication for surgery, comorbidities, ASA score, TNM stage (where relevant), presence of abscess or fistula at the time of surgery, body mass index (BMI), alcohol use and smoking history, as well as details of the surgery and a detailed description of the 30-day post-operative course.

### 2.2. Study Population

Inclusion criteria: All patients aged 16–80, who underwent ileocecectomy or right colectomy for Crohn’s disease or primary adenocarcinoma of the right colon stages 1–3, in the Department of Surgery B in Meir hospital between March 2011 and March 2016. Anastomoses were created using a standardized technique consisting of a stapled side-to-side, functional end-to-end anastomosis (GIA DST series linear stapler, Covidien, Minneapolis, MN, USA).

Exclusion criteria: Right colectomy for non-malignant polyps, stage 4 adenocarcinoma of the right colon, malignancies other than adenocarcinoma (lymphoma, melanoma, GIST, NET, etc.). Patients who underwent extended right colectomy or surgery for palliative intent were also excluded.

### 2.3. Statistical Analysis

Data were gathered into an Excel spreadsheet. Data analysis was performed using IBM SPSS Statistics for Windows, Version 25.0. Armonk, NY, USA: IBM Corp. Descriptive statistics are presented using prevalence and percentage values for categorical variables, while continuous variables are presented as means and standard deviations. Statistical analysis was performed using significance level of α = 0.05. Continuous variables were compared using a *t*-test for normally distributed variables and the Mann–Whitney–Wilcoxon test for non-normal variables. Kolmogorov–Smirnov test was used to assess normality. Categorical variables were compared using the Chi-Square or Fisher’s exact test.

Univariate Cox regression analysis was performed to evaluate the influence of various variables on 30-day complications. Inclusion into the multivariate Cox regression analysis was based on a priori selection due to clinical significance and statistical significance, as tested in the univariate Cox regression.

## 3. Results

### 3.1. Baseline Characteristics

**Figure 1:**
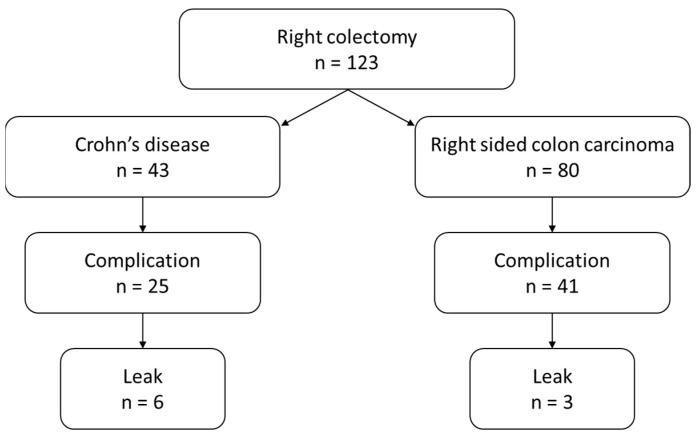
Flow chart of the study population.

A total of 123 patients were included in this study. Eighty in the right-sided colon carcinoma group versus 43 in the Crohn’s disease group (Figure 1). The mean age was 57.9 ± 18.9 years. Patients in the right-sided colon carcinoma group were older compared to the Crohn’s disease group (68.5 ± 9.9 and 38.2 ± 15.6, respectively, *p* < 0.001). Gender distribution was similar between the groups: 34/80 (42.5%) males in the right-sided colon carcinoma group and 21/43 males (48.2%) in the Crohn’s disease group (Table 1). The majority of surgeries were performed laparoscopically (82.5% and 86% for the right-sided colon carcinoma and Crohn’s disease groups, respectively, *p* = 0.6).

The right-sided colon carcinoma group had a significantly higher number of comorbidities compared to the Crohn’s disease group (5.2 ± 3.1 and 2 ± 1.4, respectively; *p* < 0.001). Moreover, ASA score distribution was significantly shifted towards lower scores in the Crohn’s disease group relative to the right-sided colon carcinoma group (Table 1). Likewise, pre-operative hemoglobin levels were significantly higher in the Crohn’s disease group compared to the right-sided colon carcinoma group (12.4 ± 1.7 and 11.5 ± 1.8, respectively; *p* = 0.02) (Table 1).

Conversely, patients in the Crohn’s disease group demonstrated significantly worse nutritional status, as mean albumin levels were 3.7 ± 0.5 in the right-sided colon carcinoma group compared with 3.4 ± 0.5 in the Crohn’s disease patients (*p* = 0.005). Likewise, 38% were underweight (defined by BMI less than 21) in the Crohn’s disease group compared to only 8% in the right-sided colon carcinoma group (*p* < 0.001).

Overall, thirty-seven percent of patients had undergone previous abdominal surgery, with 38% in the right-sided colon cancer group and 36% in the Crohn’s disease group, and no significant difference was observed between the groups.

Predictably, steroid use at the time of surgery was significantly higher among the Crohn’s disease group (46.5% (20/43) vs. 5% (4/80), *p* < 0.0001), while there was no significant difference in the rate of urgent surgeries between the groups (Table 1).

### 3.2. Post-Operative Outcomes

The overall complication rate for all patients was 53.7% (n = 66/123). The large majority of complications (75.8%) were minor, grade 1 (39.4%, n = 25) or grade 2 (36.4%, n = 26), as defined by the Clavien–Dindo classification. There was no significant difference between the two groups when comparing the overall complication rate between the right-sided colon carcinoma and Crohn’s disease groups (51.3% and 58.1%, respectively, *p* = 0.46) (Table 2). Likewise, there was no significant difference between the two groups when comparing the distribution of Clavien–Dindo scores (*p* = 0.39).

Rates of stoma formation were similar between the groups (9.9 (7/80) and 9.5% (4/43)) for the right-sided colon carcinoma and Crohn’s disease groups, respectively; *p* = 0.95). However, the leak rate was significantly higher in the Crohn’s disease group compared to the right-sided colon carcinoma group (14% (6/43) and 3.8% (3/80)), respectively, *p* = 0.04) (Table 2). Despite this, reoperation rates were not significantly different, with 6.3% (5/80) of right-sided colon carcinoma patients requiring reoperation compared to 14% (6/43) of Crohn’s disease patients (*p* = 0.15).

There was no significant difference between the groups when comparing the length of post-operative hospital stay (7.8 ± 4.7 and 9.8 ± 13.4 for the right-sided colon carcinoma and Crohn’s disease groups, respectively, *p* = 0.36) (Table 2).

### 3.3. Risk Factors for Complications

We performed a univariate binary logistic regression analysis on the entire population to assess the effect of different pre-selected variables on complication rates. This analysis showed that low pre-operative albumin levels as well as urgent surgery significantly increased the risk of post-operative complications (Table 3). Other variables, including age, gender, BMI, comorbidities, steroid use and American Society of Anesthesiologists scores did not significantly affect complication risk. On multivariate analysis, no variables were significantly associated with an increased risk of post-operative complications (data not presented).

The same analysis for the outcome of anastomotic leak did not reveal any variables that significantly increased leak risk (Table 3).

We then performed univariate and multivariate analyses for the right-sided colon carcinoma and Crohn’s disease groups separately to assess risk factors for both overall complications and anastomotic leak. No individual variable was significantly associated with either of these outcomes. (See Table 4 for multivariate analysis of risk factors for overall complications. Other analyses are not presented).

## 4. Discussion

In this study, we analyzed the data of 123 patients who underwent right colectomy at a single institution. While the majority of patients underwent surgery for right-sided colon carcinoma, one-third were patients with Crohn’s disease. When comparing baseline characteristics between the cohorts, the Crohn’s disease group appeared to be healthier as they were younger and had less comorbidities. This may theoretically confer protection against post-operative complications. However, this picture is complicated by the nutritional status of the Crohn’s disease cohort, as they had a lower BMI as well as lower albumin concentrations compared to patients with right-sided colon carcinoma. Additionally, Crohn’s disease patients had a higher steroid use at the time of surgery, which may carry with it additional risks due to medication side effects and impaired wound healing. These differences may cause difficulties in conducting a direct comparison between the two groups. However, in our opinion, these characteristics are integral to the differences between the two diseases and, therefore, should remain as part of the analysis without performing statistical manipulations to equalize these variables. Furthermore, in the regression analysis, age, comorbidities, American Society of Anesthesiologists scores and steroid use were not found to contribute to the risk of surgical complications.

The overall complication rate for all patients was 53.66% (66/123). While this number appears high, it is worth noting that a large majority of complications (75.8%) were minor (Clavien–Dindo grades 1 and 2). The high complication rate may also be explained by the fact that the evaluation and classification of post-operative complications were made by an impartial medical student, not part of the Department, and therefore less affected by “bias”. Furthermore, leak rates were not different from those previously reported in the literature [7,14].

There was no significant difference between the groups when comparing the overall complication rate, the severity of complications (as measured by the distribution of the Clavien–Dindo scores) and the length of post-operative hospital stay. While previous studies have shown that older age and the number of comorbidities are independent risk factors for worse post-operative outcomes [7,12,14,15,16,18], we hypothesized that Crohn’s disease patients would have higher complication rates due to the underlying diseased state of the bowel, as well as steroid use and malnutrition. The lack of difference in complication rates may be explained by the fact that patients in the Crohn’s disease group were younger and had less comorbidities conferring on them a measure of protection compared to the right-sided colon carcinoma cohort. Furthermore, malnourished patients with Crohn’s disease underwent prehabilitation using exclusive enteral nutrition (EEN) or parenteral nutrition (PN) combined with attempts to decrease steroid use in order to minimize these risk factors. Any intra-abdominal abscesses were also treated pre-operatively.

This is supported by previous studies, which have shown that optimal preparation of Crohn’s patients prior to surgery significantly improves post-operative outcomes [3,4,9,10]. Given these findings, surgery for Crohn’s disease may not be as inherently risky as hypothesized when compared to other colorectal surgeries if patients are prepared accordingly.

Although overall complication rates were similar, when examining anastomotic leaks specifically, patients in the Crohn’s disease cohort had worse outcomes. The higher leak rate may be explained by several factors: Crohn’s disease patients have an impaired wound healing mechanism due to their underlying disease, which may affect the healing of the anastomosis. Moreover, although an effort was made in order to optimize Crohn’s disease patients’ metabolic condition prior to surgery, patients in this group had significantly poorer nutritional status (as measured by albumin levels as well as BMI), and a significant number of them were receiving steroid treatment in the pre-operative period—all of which are known risk factors associated with a higher leak rate [4,7,9,10,12,15,16,17,20]. Another important factor to consider regarding complication risk is other medical treatments, specifically neo-adjuvant chemotherapy in the colorectal cancer cohort (although no patient in our group received treatment prior to surgery or within 30 days post-op) and immunomodulatory therapy in the Crohn’s disease cohort. Furthermore, the inherent differences between Crohn’s disease and right-sided colon cancer may also affect complication rates. Whereas Crohn’s disease is a chronic disease possibly involving multiple foci of disease, right-sided colon cancer is usually localized to one area of colon and is operated on shortly after diagnosis. Unfortunately, our study population was underpowered to adequately assess the impact of these potentially important variables. Additional planned studies will hopefully give clearer answers to these questions.

Univariate regression analysis of the entire population revealed low albumin levels and urgent surgery as significant risk factors for surgical complications. On multivariate analysis, however, neither variable reached statistical significance. As hypoalbuminemia is a risk factor for surgical complications, and patients with Crohn’s disease have an increased likelihood of being hypoalbuminemic, we proceeded to conduct univariate and multivariate analyses using the same variables for each cohort separately. This analysis did not reveal any statistically significant risk factors, possibly due to the study being underpowered for this subgroup analysis.

### Limitations

Our study has several limitations. First, this is a retrospective analysis of electronic medical records from a single medical center. Therefore, selection bias and unidentified confounders may have an impact on the results. Furthermore, the cohort sizes were not equal, possibly introducing an additional bias. Second, data retrieval was performed by a medical student and not a surgeon. This may have contributed to the relatively high complication rate dominated by low-grade complications, but may have reduced bias; however, some minor disruptions of normal post-op progression may be coded as complications by a non-surgeon. Third, the study may be underpowered to conduct more advanced sub-group analyses, such as the effects of treatments other than steroids (i.e., chemotherapy or Tumor Necrosis Factor inhibitors). This limitation is inherent to the retrospective nature of this study. Additionally, it is noteworthy that due to the multiplicity of variables, statistical artifacts may have been introduced from multiple *p*-value calculations (i.e., when comparing leak rates).

## 5. Conclusions

This study has shown that the overall post-operative complication rate after a right colectomy performed due to Crohn’s disease is similar to that in the case of adenocarcinoma of the right colon. Moreover, there was no statistically significant difference between the groups when comparing the complication severity and the length of post-operative hospital stay. However, Crohn’s disease patients had a significantly higher anastomotic leak rate. This may be due to impaired nutritional status, stressing the importance of adequate metabolic prehabilitation. Additional studies, with a larger sample size, are needed in order to further investigate these findings.

## Figures and Tables

**Table 1:** Baseline characteristics of all patients at hospital admission. ASA—American Society of Anesthesiologists’ (ASA) classification of physical health; WBC—white blood cells; BMI—body mass index.

Variable	Overall	CRC	CD	*p*-Value
Patients [n (%)]	123	80 (65%)	43 (35%)	
Age [years]	57.9 ± 18.9	68.5 ± 9.9	38.2 ± 15.6	<0.001
Gender [male, n (%)]	55 (65)	34 (42.5)	21 (48.2)	0.57
ASA [n (%)]				0.005
ASA 1	7 (5.7)	5 (6.3)	2 (4.7)	
ASA 2	81 (65.9)	44 (55)	37 (86)	
ASA 3	31 (25.2)	27 (33.8)	4 (9.3)	
ASA 4	4 (3.3)	4 (5)	0 (0)	
Comorbidities	4.1 ± 3.1	5.2 ± 3.1	2 ± 1.4	<0.001
Tobacco [users, n (%)]	28 (22.8)	16 (20)	12 (27.9)	0.319
Steroids [yes, n (%)]	24 (19.5)	4 (5)	20 (46.5)	<0.001
BMI (kg/m^2^)	25.5 ± 5.5	27.6 ± 5.2	21.9 ± 3.9	<0.001
Albumin (g/dL)	3.6 ± 0.5	3.7 ± 0.5	3.4 ± 0.5	0.005
Previous surgery [n (%)]	45 (37)	30 (38)	15 (36)	0.85
Hemoglobin (g/dL)	11.8 ± 1.8	11.5 ± 1.8	12.4 ± 1.7	0.02
WBC (K/µL)	8.5 ± 3.6	8 ± 2.9	9.2 ± 4.5	0.08
Urgent [yes, n (%)]	22 (17.9)	11 (13.6)	11 (25.6)	0.1

**Table 2:** Thirty-day outcomes. LOS—length of stay, CD—Clavian–Dindo.

Variable	Overall	CRC	CD	*p*-Value
LOS [days, mean ± SD]	8.5 ± 8.8	7.8 ± 4.7	9.8 ± 13.4	0.36
Stoma [n (%)]	11 (9.7)	7 (9.9)	4 (9.5)	0.95
Complication [n (%)]	66 (53.7)	41 (51.3)	25 (58.1)	0.46
Leak [n (%)]	9 (7.4)	3 (3.8)	6 (14)	0.04
Conversion [n (%)]	12 (9.8)	5 (6.3)	7 (16.3)	0.08
30 day reoperation [n (%)]	11 (8.9)	5 (6.3)	6 (14)	0.15
30 day mortality [n (%)]	2 (1.6)	2 (2.5)	0 (0)	0.29
Clavian–Dindo classification				0.39
CD-1	26 (39.4)	18 (43.9)	8 (32)	
CD-2	24 (36.4)	14 (34.1)	10 (40)	
CD-3	7 (10.6)	4 (9.8)	3 (12)	
CD-4	7 (10.6)	3 (7.3)	4 (16)	
CD-5	2 (3.0)	2 (4.9)	0 (0)	

**Table 3:** Univariate analysis of risk factors for surgical complication and leak. OR—odds ratio; BMI—body mass index; ASA—American Society of Anesthesiologists physical status classification system. * indicates statistically significant association.

	Complication			Leak		
Variable	OR	95% Confidence Limits		OR	95% Confidence Limits	
Age	0.99	0.97	1.01	0.98	0.95	1.01
Gender (female)	0.94	0.46	1.91	0.38	0.09	1.61
BMI (kg/m^2^)	0.95	0.89	1.03	0.91	0.78	1.07
Comorbidities	0.90	0.80	1.02	0.95	0.75	1.20
Steroid use	1.03	0.42	2.51	1.45	0.58	3.61
Tobacco	1.45	0.62	3.42	1.10	0.47	2.58
Albumin (g/dL)	0.34 *	0.14	0.83	0.33	0.09	1.3
Hemoglobin (g/dL)	1.02	0.83	1.25	1.03	0.69	1.52
Urgent	3.61 *	1.24	10.53	0.39	0.15	1.02
ASA (ASA-1 ref.)						
ASA-2	1.33	0.11	15.7	1	0	>10,000
ASA-3	1.19	0.16	8.86	>1000	0	>10,000
ASA-4	1.07	0.13	8.56	>1000	0	>10,000

**Table 4:** Multivariate analysis of risk factors for surgical complication. OR—odds ratio; BMI—body mass index; ASA—American Society of Anesthesiologists physical status classification system.

	CRC			CD		
Variable	OR	95% Confidence Limits		OR	95% Confidence Limits	
Age	0.91	0.79	1.04	0.98	0.87	1.10
Gender (female)	0.81	0.16	4.02	1.15	0.07	19.25
BMI (kg/m^2^)	1.024	0.86	1.22	0.82	0.61	1.12
Comorbidities	0.81	0.56	1.17	1.15	0.42	3.12
Steroid use	0.95	0.04	21.26	32.05	0.61	1694
Tobacco	0.58	0.05	6.37	4.37	0.14	133.24
Albumin (g/dL)	0.56	0.04	6.86	0.09	0.00	11.38
Hemoglobin (g/dL)	1.18	0.72	1.93	2.74	0.59	12.67
Urgent	0.43	0.02	9.71	0.05	0.00	1.53
ASA (ASA-1 ref.)						
ASA-2	700	0.00	>100	0.00	0.00	>1000
ASA-3	6500	0.01	>10,000	1.9	0.03	139.17
ASA-4	12,000	0.01	>100,000	N/A	N/A	N/A

## Data Availability

The data presented in this study are available on reasonable request from the corresponding author.
